# Diaqua­bis­(2-oxo-2*H*-chromene-3-carboxyl­ato-κ^2^ *O* ^2^,*O* ^3^)cadmium

**DOI:** 10.1107/S1600536810053523

**Published:** 2010-12-24

**Authors:** Yue Cui, Qian Gao, Huan-Huan Wang, Lin Wang, Ya-Bo Xie

**Affiliations:** a College of Environmental and Energy Engineering, Beijing University of Technology, Beijing 100124, People’s Republic of China

## Abstract

In the title mononuclear cadmium complex, [Cd(C_10_H_5_O_4_)_2_(H_2_O)_2_], the Cd^II^ atom, located on a crystallographic inversion center, exhibits a slightly distorted octa­hedral geometry and is six-coordinated by two O atoms from water mol­ecules in the axial positions and four O atoms from two deprotonated coumarin-3-carb­oxy­lic acid ligands in the equatorial plane. Angles around the Cd^II^ atom vary between 81.00 (5) and 99.00 (0)°. The Cd—O bond lengths vary between 2.1961 (13) and 2.3360 (13) Å. O—H⋯O hydrogen bonds between the H atoms of coordinated water mol­ecules and the O atoms of carboxyl­ate groups link the complex mol­ecules into layers parallel to the *ab* plane.

## Related literature

For background to topological networks, see: Lin *et al.* (2010 ▶). For applications of self-assembling systems with organic ligands containing O donors, see: Bischof *et al.* (2010 ▶); Chen *et al.* (2008 ▶); Ghoshal *et al.* (2007 ▶); Li & Zhou (2009 ▶). For related structures, see: Georgieva *et al.* (2007 ▶); Li *et al.* (2009 ▶).
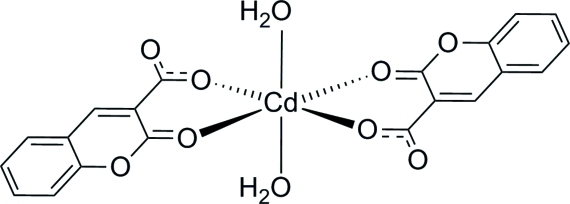


## Experimental

### 

#### Crystal data

[Cd(C_10_H_5_O_4_)_2_(H_2_O)_2_]
*M* *_r_* = 526.72
Triclinic, 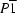

*a* = 6.6736 (13) Å
*b* = 6.8838 (14) Å
*c* = 10.477 (2) Å
α = 93.37 (3)°
β = 91.46 (3)°
γ = 112.07 (3)°
*V* = 444.68 (15) Å^3^
*Z* = 1
Mo *K*α radiation
μ = 1.29 mm^−1^
*T* = 110 K
0.20 × 0.15 × 0.15 mm

#### Data collection

Bruker APEXII CCD diffractometer
Absorption correction: multi-scan (*SADABS*; Sheldrick, 2008*a* ▶) *T*_min_ = 0.793, *T*_max_ = 0.824
2812 measured reflections
2040 independent reflections
2033 reflections with *I* > 2σ(*I*)
*R*_int_ = 0.009

#### Refinement

*R*[*F* ^2^ > 2σ(*F* ^2^)] = 0.017
*wR*(*F* ^2^) = 0.045
*S* = 1.12
2040 reflections
142 parameters
H-atom parameters constrained
Δρ_max_ = 0.42 e Å^−3^
Δρ_min_ = −0.44 e Å^−3^

### 

Data collection: *APEX2* (Bruker, 2008 ▶); cell refinement: *SAINT* (Bruker, 2008 ▶); data reduction: *SAINT*; program(s) used to solve structure: *SHELXS97* (Sheldrick, 2008*b* ▶); program(s) used to refine structure: *SHELXL97* (Sheldrick, 2008*b* ▶); molecular graphics: *SHELXTL* (Sheldrick, 2008*b* ▶); software used to prepare material for publication: *SHELXL97*.

## Supplementary Material

Crystal structure: contains datablocks global, I. DOI: 10.1107/S1600536810053523/zl2335sup1.cif

Structure factors: contains datablocks I. DOI: 10.1107/S1600536810053523/zl2335Isup2.hkl

Additional supplementary materials:  crystallographic information; 3D view; checkCIF report

## Figures and Tables

**Table 1:** Hydrogen-bond geometry (Å, °)

*D*—H⋯*A*	*D*—H	H⋯*A*	*D*⋯*A*	*D*—H⋯*A*
O1*W*—H1*WA*⋯O4^i^	0.85	1.90	2.6877 (18)	153
O1*W*—H1*WB*⋯O4^ii^	0.85	1.94	2.721 (2)	153

Symmetry codes: (i) 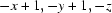; (ii) 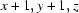.

**Fig. 1.**
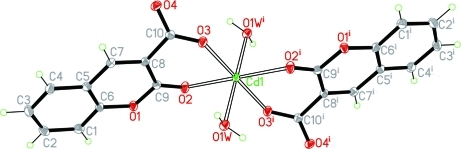
The molecular structure of the title compound with displacement ellipsoids drawn at the 50% probability level for non-hydrogen atoms, hydrogen atoms are shown as small circles of arbitrary radius. [Symmetry code: i = -x + 2, -y + 1, -z].

**Fig. 2.**
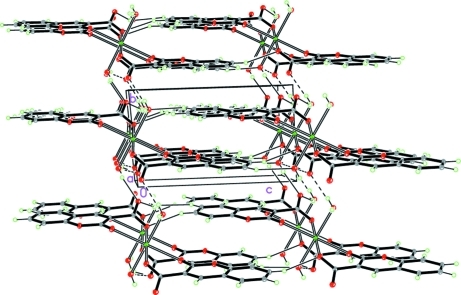
Partial packing view of title compound, showing the formation of network built from hydrogen bonds.

## supplementary crystallographic information

### Comment

In the past decades, much attention has been paid to the design and synthesis of self-assembling metal complex systems with organic ligands containing O donors due to their fascinating structural diversity (Lin *et al.*, 2010) and potential applications in the areas of catalysis (Bischof *et al.*, 2010), magnetism (Ghoshal *et al.*, 2007), gas adsorption (Li & Zhou, 2009), and luminescence (Chen *et al.*, 2008). Coumarin-3-carboxylic acid is such a ligand and complexes containing it have been reported (Georgieva *et al.*, 2007). Herein, we report the synthesis and crystal structure of a new mononuclear cadmium complex coordinated by coumarin-3-carboxylic acid.

The molecule of the title mononuclear cadmium(II) complex, [Cd(C_10_H_5_O_4_)_2_(H_2_O)_2_], occupies a special position with the metal center being located on a crystallographic inversion center. Each Cd^II^ atom exhibits a slightly distorted octahedral geometry and is six-coordinated by two O atoms from water molecules in the axial positions and four O atoms from two deprotonated coumarin-3-carboxylic acid ligands in the equatorial plane. Angles around the Cd^II^ atom vary between 81.02 (6)° and 98.98 (8)°. The Cd—O bond distances between the Cd^II^ atom and the O atoms vary between 2.196 (2) and 2.336 (2) Å, all of which are comparable to those reported for other cadmium-oxygen donor complexes (e.g., Li *et al.*, 2009). The (C1C2C3C4C5C6) ring and the (C6C5C7C8C9O1) ring are almost coplanar, and the dihedral angles is 1.673 (5)°. The dihedral angle between The C8C9C10O2 plane and the O2O3Cd1 plane is 28.541 (7)°. O-H···O hydrogen bonds between the hydrogen atoms of coordinated water molecules and the O atoms of carboxyl groups joins the complexes into two-dimensional layers parallel the *ab* plane (Table 1, Fig. 2).

### Experimental

The title complex was synthesized by carefully layering a solution of Cd(NO_3_)_2_.4H_2_O (30.8 mg, 0.1 mmol) in ethanol (10 ml) on top of a solution of coumarin-3-carboxylic acid (19.0 mg, 0.1 mmol) and LiOH (8.4 mg, 0.2 mmol) in H_2_O (10 ml) in a test-tube. After about one month at room temperature, colorless block-shaped single crystals suitable for X-ray investigation appeared at the boundary between the ethanol solution and the water layer with a yield of 23% (12.1 mg). Decomposition temperature: near 573K. FT-IR (KBr, cm^-1^): 636, 748, 777, 1183, 1281, 1394, 1450, 1562, 1604, 1674.

### Refinement

Carbon H atoms were placed geometrically (C—H = 0.93 Å) and treated as riding with *U*_iso(H)_ = 1.2*U*_eq_(C). Water H atoms were located in calculated positions and treated in the subsequent refinement as riding atoms, with O-H = 0.85 Å and *U*_iso_(H) = 1.5*U*_eq_(O).

### Crystal data

**Table:**

[Cd(C_10_H_5_O_4_)_2_(H_2_O)_2_]	*Z* = 1
*M**_r_* = 526.72	*F*(000) = 262
Triclinic, *P*1	*D*_x_ = 1.967 Mg m^−^^3^
Hall symbol: -P 1	Mo *K*α radiation, λ = 0.71073 Å
*a* = 6.6736 (13) Å	Cell parameters from 2723 reflections
*b* = 6.8838 (14) Å	θ = 3.2–28.3°
*c* = 10.477 (2) Å	µ = 1.29 mm^−^^1^
α = 93.37 (3)°	*T* = 110 K
β = 91.46 (3)°	Block, colorless
γ = 112.07 (3)°	0.20 × 0.15 × 0.15 mm
*V* = 444.68 (15) Å^3^	

### Data collection

**Table:**

Bruker APEXII CCD diffractometer	2040 independent reflections
Radiation source: fine-focus sealed tube	2033 reflections with *I* > 2σ(*I*)
graphite	*R*_int_ = 0.009
φ and ω scans	θ_max_ = 28.3°, θ_min_ = 3.2°
Absorption correction: multi-scan (*SADABS*; Sheldrick, 2008*a*)	*h* = −8→8
*T*_min_ = 0.793, *T*_max_ = 0.824	*k* = −8→8
2812 measured reflections	*l* = −13→6

### Refinement

**Table:**

Refinement on *F*^2^	Primary atom site location: structure-invariant direct methods
Least-squares matrix: full	Secondary atom site location: difference Fourier map
*R*[*F*^2^ > 2σ(*F*^2^)] = 0.017	Hydrogen site location: inferred from neighbouring sites
*wR*(*F*^2^) = 0.045	H-atom parameters constrained
*S* = 1.12	*w* = 1/[σ^2^(*F*_o_^2^) + (0.0217*P*)^2^ + 0.3208*P*] where *P* = (*F*_o_^2^ + 2*F*_c_^2^)/3
2040 reflections	(Δ/σ)_max_ < 0.001
142 parameters	Δρ_max_ = 0.42 e Å^−^^3^
0 restraints	Δρ_min_ = −0.44 e Å^−^^3^

### Special details

**Table:**

Geometry. All esds (except the esd in the dihedral angle between two l.s. planes) are estimated using the full covariance matrix. The cell esds are taken into account individually in the estimation of esds in distances, angles and torsion angles; correlations between esds in cell parameters are only used when they are defined by crystal symmetry. An approximate (isotropic) treatment of cell esds is used for estimating esds involving l.s. planes.
Refinement. Refinement of F^2^ against ALL reflections. The weighted R-factor wR and goodness of fit S are based on F^2^, conventional R-factors R are based on F, with F set to zero for negative F^2^. The threshold expression of F^2^ > 2sigma(F^2^) is used only for calculating R-factors(gt) etc. and is not relevant to the choice of reflections for refinement. R-factors based on F^2^ are statistically about twice as large as those based on F, and R- factors based on ALL data will be even larger.

### Fractional atomic coordinates and isotropic or equivalent isotropic displacement parameters (Å^2^)

**Table:**

	*x*	*y*	*z*	*U*_iso_*/*U*_eq_	
Cd1	1.0000	0.5000	0.0000	0.01268 (6)	
O1	0.79598 (18)	0.30467 (18)	0.37932 (11)	0.0144 (2)	
O1W	1.00099 (19)	0.79679 (19)	0.10974 (11)	0.0163 (2)	
H1WA	0.8758	0.7921	0.0875	0.024*	
H1WB	1.1256	0.8837	0.0939	0.024*	
O2	0.93465 (18)	0.33456 (19)	0.19160 (11)	0.0165 (2)	
O3	0.64486 (18)	0.35849 (19)	−0.01845 (11)	0.0161 (2)	
O4	0.31702 (18)	0.12595 (19)	0.01275 (11)	0.0164 (2)	
C1	0.6758 (3)	0.2758 (3)	0.59042 (16)	0.0170 (3)	
H1A	0.8180	0.2975	0.6218	0.020*	
C2	0.5088 (3)	0.2453 (3)	0.67327 (16)	0.0186 (3)	
H2A	0.5371	0.2462	0.7627	0.022*	
C3	0.2996 (3)	0.2132 (3)	0.62646 (16)	0.0192 (3)	
H3A	0.1869	0.1917	0.6841	0.023*	
C4	0.2562 (3)	0.2127 (3)	0.49686 (16)	0.0162 (3)	
H4A	0.1144	0.1926	0.4657	0.019*	
C5	0.4221 (3)	0.2421 (2)	0.41095 (15)	0.0137 (3)	
C6	0.6282 (3)	0.2734 (2)	0.46024 (15)	0.0138 (3)	
C7	0.3889 (3)	0.2351 (2)	0.27516 (15)	0.0131 (3)	
H7A	0.2487	0.2123	0.2398	0.016*	
C8	0.5526 (3)	0.2604 (2)	0.19545 (15)	0.0123 (3)	
C9	0.7689 (3)	0.3026 (2)	0.24937 (15)	0.0127 (3)	
C10	0.5040 (3)	0.2474 (2)	0.05228 (15)	0.0127 (3)	

### Atomic displacement parameters (Å^2^)

**Table:**

	*U*^11^	*U*^22^	*U*^33^	*U*^12^	*U*^13^	*U*^23^
Cd1	0.00892 (8)	0.01495 (9)	0.01174 (9)	0.00156 (6)	0.00160 (5)	0.00172 (5)
O1	0.0121 (5)	0.0186 (6)	0.0118 (5)	0.0048 (4)	0.0005 (4)	0.0029 (4)
O1W	0.0119 (5)	0.0180 (6)	0.0174 (6)	0.0038 (4)	0.0014 (4)	0.0014 (4)
O2	0.0116 (5)	0.0228 (6)	0.0155 (6)	0.0062 (5)	0.0023 (4)	0.0057 (4)
O3	0.0111 (5)	0.0209 (6)	0.0138 (5)	0.0027 (5)	0.0006 (4)	0.0037 (4)
O4	0.0109 (5)	0.0179 (6)	0.0164 (5)	0.0010 (4)	−0.0018 (4)	0.0020 (4)
C1	0.0190 (8)	0.0147 (7)	0.0158 (8)	0.0048 (6)	−0.0011 (6)	0.0018 (6)
C2	0.0285 (9)	0.0148 (7)	0.0119 (7)	0.0072 (7)	0.0018 (6)	0.0015 (6)
C3	0.0242 (9)	0.0162 (8)	0.0168 (8)	0.0068 (7)	0.0083 (6)	0.0019 (6)
C4	0.0158 (7)	0.0147 (7)	0.0176 (8)	0.0048 (6)	0.0036 (6)	0.0021 (6)
C5	0.0155 (7)	0.0107 (7)	0.0140 (7)	0.0037 (6)	0.0016 (6)	0.0012 (5)
C6	0.0155 (7)	0.0108 (7)	0.0143 (7)	0.0037 (6)	0.0036 (6)	0.0018 (5)
C7	0.0116 (7)	0.0126 (7)	0.0147 (7)	0.0041 (6)	0.0005 (6)	0.0020 (5)
C8	0.0119 (7)	0.0117 (7)	0.0129 (7)	0.0037 (6)	0.0005 (5)	0.0018 (5)
C9	0.0132 (7)	0.0118 (7)	0.0124 (7)	0.0037 (6)	−0.0005 (5)	0.0020 (5)
C10	0.0114 (7)	0.0138 (7)	0.0133 (7)	0.0055 (6)	−0.0002 (5)	0.0006 (5)

### Geometric parameters (Å, °)

**Table:**

Cd1—O3^i^	2.1961 (13)	C1—C2	1.391 (2)
Cd1—O3	2.1961 (13)	C1—H1A	0.9500
Cd1—O1W	2.2824 (13)	C2—C3	1.400 (3)
Cd1—O1W^i^	2.2824 (13)	C2—H2A	0.9500
Cd1—O2^i^	2.3360 (13)	C3—C4	1.381 (2)
Cd1—O2	2.3360 (13)	C3—H3A	0.9500
O1—C9	1.3673 (19)	C4—C5	1.407 (2)
O1—C6	1.3810 (19)	C4—H4A	0.9500
O1W—H1WA	0.8500	C5—C6	1.390 (2)
O1W—H1WB	0.8500	C5—C7	1.430 (2)
O2—C9	1.227 (2)	C7—C8	1.357 (2)
O3—C10	1.257 (2)	C7—H7A	0.9500
O4—C10	1.256 (2)	C8—C9	1.453 (2)
C1—C6	1.390 (2)	C8—C10	1.517 (2)
			
O3^i^—Cd1—O3	180.0	C3—C2—H2A	119.6
O3^i^—Cd1—O1W	87.17 (5)	C4—C3—C2	120.30 (16)
O3—Cd1—O1W	92.83 (5)	C4—C3—H3A	119.8
O3^i^—Cd1—O1W^i^	92.83 (5)	C2—C3—H3A	119.8
O3—Cd1—O1W^i^	87.17 (5)	C3—C4—C5	120.02 (16)
O1W—Cd1—O1W^i^	180.00 (5)	C3—C4—H4A	120.0
O3^i^—Cd1—O2^i^	81.00 (5)	C5—C4—H4A	120.0
O3—Cd1—O2^i^	99.00 (5)	C6—C5—C4	118.38 (15)
O1W—Cd1—O2^i^	91.77 (5)	C6—C5—C7	118.06 (14)
O1W^i^—Cd1—O2^i^	88.23 (5)	C4—C5—C7	123.54 (15)
O3^i^—Cd1—O2	99.00 (5)	O1—C6—C5	120.21 (14)
O3—Cd1—O2	81.00 (5)	O1—C6—C1	117.23 (15)
O1W—Cd1—O2	88.23 (5)	C5—C6—C1	122.57 (15)
O1W^i^—Cd1—O2	91.77 (5)	C8—C7—C5	121.68 (15)
O2^i^—Cd1—O2	180.00 (3)	C8—C7—H7A	119.2
C9—O1—C6	122.72 (13)	C5—C7—H7A	119.2
Cd1—O1W—H1WA	100.6	C7—C8—C9	119.32 (14)
Cd1—O1W—H1WB	100.5	C7—C8—C10	118.64 (14)
H1WA—O1W—H1WB	130.4	C9—C8—C10	122.04 (14)
C9—O2—Cd1	123.19 (11)	O2—C9—O1	114.46 (14)
C10—O3—Cd1	132.88 (11)	O2—C9—C8	127.61 (15)
C6—C1—C2	117.96 (16)	O1—C9—C8	117.92 (14)
C6—C1—H1A	121.0	O4—C10—O3	124.02 (15)
C2—C1—H1A	121.0	O4—C10—C8	115.83 (14)
C1—C2—C3	120.77 (15)	O3—C10—C8	120.11 (14)
C1—C2—H2A	119.6		
			
O3^i^—Cd1—O2—C9	151.16 (12)	C2—C1—C6—C5	0.0 (2)
O3—Cd1—O2—C9	−28.84 (12)	C6—C5—C7—C8	−0.7 (2)
O1W—Cd1—O2—C9	64.31 (13)	C4—C5—C7—C8	−178.75 (15)
O1W^i^—Cd1—O2—C9	−115.69 (13)	C5—C7—C8—C9	−2.1 (2)
O1W—Cd1—O3—C10	−87.46 (15)	C5—C7—C8—C10	178.98 (14)
O1W^i^—Cd1—O3—C10	92.54 (15)	Cd1—O2—C9—O1	−149.14 (10)
O2^i^—Cd1—O3—C10	−179.70 (15)	Cd1—O2—C9—C8	32.0 (2)
O2—Cd1—O3—C10	0.30 (15)	C6—O1—C9—O2	179.45 (13)
C6—C1—C2—C3	0.0 (2)	C6—O1—C9—C8	−1.5 (2)
C1—C2—C3—C4	−0.4 (3)	C7—C8—C9—O2	−177.95 (16)
C2—C3—C4—C5	0.7 (2)	C10—C8—C9—O2	0.9 (3)
C3—C4—C5—C6	−0.7 (2)	C7—C8—C9—O1	3.2 (2)
C3—C4—C5—C7	177.35 (15)	C10—C8—C9—O1	−177.92 (13)
C9—O1—C6—C5	−1.3 (2)	Cd1—O3—C10—O4	−156.56 (12)
C9—O1—C6—C1	178.95 (14)	Cd1—O3—C10—C8	25.5 (2)
C4—C5—C6—O1	−179.43 (14)	C7—C8—C10—O4	−31.7 (2)
C7—C5—C6—O1	2.4 (2)	C9—C8—C10—O4	149.44 (15)
C4—C5—C6—C1	0.3 (2)	C7—C8—C10—O3	146.42 (16)
C7—C5—C6—C1	−177.83 (14)	C9—C8—C10—O3	−32.5 (2)
C2—C1—C6—O1	179.78 (14)		

Symmetry codes: (i) −*x*+2, −*y*+1, −*z*.

### Hydrogen-bond geometry (Å, °)

**Table:**

*D*—H···*A*	*D*—H	H···*A*	*D*···*A*	*D*—H···*A*
O1W—H1WA···O4^ii^	0.85	1.90	2.6877 (18)	153
O1W—H1WB···O4^iii^	0.85	1.94	2.721 (2)	153

Symmetry codes: (ii) −*x*+1, −*y*+1, −*z*; (iii) *x*+1, *y*+1, *z*.

## References

[bb1] Bischof, S. M., Ess, D. H., Meier, S. K., Oxgaard, J., Nielsen, R. J., Bhalla, G., Goddard, W. A. & Periana, R. A. (2010). *Organometallics*, **29**, 742–756.

[bb2] Bruker (2008). *APEX2* and *SAINT* Bruker AXS Inc., Madison, Wisconsin, USA.

[bb3] Chen, L. F., Li, Z. J., Qin, Y. Y., Cheng, J. K. & Yao, Y. G. (2008). *J. Mol. Struct.* **892**, 278–282.

[bb4] Georgieva, I., Trendafilova, N., Aquino, A. J. A. & Lischka, H. (2007). *Inorg. Chem.* **46**, 10926–10936. 10.1021/ic7016616 17990875

[bb5] Ghoshal, D., Ghosh, A. K., Mostafa, G., Ribas, J. & Chaudhuri, N. R. (2007). *Inorg. Chim. Acta*, **360**, 1771–1775.

[bb6] Li, N., Gou, L., Hu, H. M., Chen, S. H., Chen, X. L., Wang, B. W., Wu, Q. R., Yang, M. L. & Xue, G. L. (2009). *Inorg. Chim. Acta*, **362**, 3475–3483.

[bb7] Li, J. R. & Zhou, H. C. (2009). *Angew. Chem. Int. Ed* **48**, 1–5.

[bb8] Lin, J. D., Long, X. F., Lin, P. & Du, S. W. (2010). *Cryst. Growth Des.* **10**, 146–157.

[bb9] Sheldrick, G. M. (2008*a*). *SADABS* University of Göttingen, Germany.

[bb10] Sheldrick, G. M. (2008*b*). *Acta Cryst.* A**64**, 112–122. 10.1107/S0108767307043930 18156677

